# Temporal radiographic changes in COVID-19 patients: relationship to disease severity and viral clearance

**DOI:** 10.1038/s41598-020-66895-w

**Published:** 2020-06-24

**Authors:** Xiaofan Liu, Hong Zhou, Yilu Zhou, Xiaojun Wu, Yang Zhao, Yang Lu, Weijun Tan, Mingli Yuan, Xuhong Ding, Jinjing Zou, Ruiyun Li, Hailing Liu, Rob M. Ewing, Yi Hu, Hanxiang Nie, Yihua Wang

**Affiliations:** 1 Department of Pulmonary and Critical Care Medicine, The Central Hospital of Wuhan, Tongji Medical College, Huazhong University of Science and Technology, Wuhan, Hubei China; 2 Biological Sciences, Faculty of Environmental and Life Sciences, University of Southampton, Southampton, SO17 1BJ UK; 3 Institute for Life Sciences, University of Southampton, Southampton, SO17 1BJ UK; 4 Department of Respiratory & Critical Medicine, Renmin Hospital of Wuhan University, Wuhan, 430060 Hubei China; 5 NIHR Southampton Biomedical Research Centre, University Hospital Southampton, Southampton, SO16 6YD UK

**Keywords:** Diseases, Health care, Medical research, Risk factors, Signs and symptoms

## Abstract

COVID-19 is “public enemy number one” and has placed an enormous burden on health authorities across the world. Given the wide clinical spectrum of COVID-19, understanding the factors that can predict disease severity will be essential since this will help frontline clinical staff to stratify patients with increased confidence. To investigate the diagnostic value of the temporal radiographic changes, and the relationship to disease severity and viral clearance in COVID-19 patients. In this retrospective cohort study, we included 99 patients admitted to the Renmin Hospital of Wuhan University, with laboratory confirmed moderate or severe COVID-19. Temporal radiographic changes and viral clearance were explored using appropriate statistical methods. Radiographic features from HRCT scans included ground-glass opacity, consolidation, air bronchogram, nodular opacities and pleural effusion. The HRCT scores (peak) during disease course in COVID-19 patients with severe pneumonia (median: 24.5) were higher compared to those with pneumonia (median: 10) (p = 3.56 × 10 ^−12^), with more frequency of consolidation (p = 0.025) and air bronchogram (p = 7.50 × 10^−6^). The median values of days when the peak HRCT scores were reached in pneumonia or severe pneumonia patients were 12 *vs*. 14, respectively (p = 0.048). Log-rank test and Spearman’s Rank-Order correlation suggested temporal radiographic changes as a valuable predictor for viral clearance. In addition, follow up CT scans from 11 pneumonia patients showed full recovery. Given the values of HRCT scores for both disease severity and viral clearance, a standardised HRCT score system for COVID-19 is highly demanded.

## Introduction

In December 2019, a pneumonia of unknown cause was linked to a seafood wholesale market in Wuhan, China. A novel coronavirus^1,2^, severe acute respiratory syndrome coronavirus 2 (SARS-CoV-2; previously known as 2019-nCoV), was isolated from these patients^3,4^, which was later designated coronavirus disease 2019 (COVID-19) by WHO. The COVID-19 outbreak was declared a Public Health Emergency of International Concern on 30 January 2020 and was characterized as a pandemic on 11 March 2020. It has placed an enormous burden on health authorities across the world, including more than 200 countries and territories.

The clinical spectrum of COVID-19 ranges from mild disease with non-specific signs and symptoms of acute respiratory illness, to severe pneumonia with respiratory failure and septic shock. There have also been reports of asymptomatic infection with COVID-19^5–7^. Given the wide clinical spectrum of COVID-19, a key challenge faced by frontline clinical staff is prioritisation of stretched resources as well as predicting prognosis. Thus, there is a critical need for robust risk assessment for clinical management. Older age (>65 years) and comorbidities have been reported as risk factors for death^8,9^. Zhou and colleagues reported older age, high Sequential Organ Failure Assessment (SOFA) score, and d-dimer greater than 1 μg/L as potential risk factors for mortality^7^. However, to our knowledge, at present, there is limited standardised method to predict which infected patient will remain moderately symptomatic and which will progress to more severe disease as well as viral clearance.

Here, we present details of 99 patients admitted to the Renmin Hospital of Wuhan University (Hubei, China), with laboratory confirmed moderate or severe COVID-19. We aim to describe the temporal radiographic changes, and the relationship to disease severity and viral clearance in COVID-19 patients.

## Materials and Methods

### Study design and participants

All methods were carried out in accordance with relevant guidelines and regulations. This retrospective study was approved by the Ethics Committee of Renmin Hospital of Wuhan University, Hubei, China (No. WDRY2020-K124), and the requirement for informed consent was waived by the Ethics Committee due to a public health outbreak investigation.

We identified consecutive patients with moderate or severe COVID-19 discharged from the general wards of Renmin Hospital of Wuhan University between 5 February 2020 to 14 March 2020. All patients had been diagnosed with COVID-19 according to WHO interim guidance^7,10,11^. On admission, patients are classified into the following 4 severity stages: mild, moderate, severe and critical, as defined by the COVID-19 Chinese guidelines document (version 7). *Mild* cases are defined by mild symptoms and no evidence of pneumonia while *moderate* cases are defined by fever, respiratory tract and other symptoms, and radiological evidence of pneumonia. *Severe* cases meet any of the following criteria: respiratory rate ≥30 breaths/min; oxygen saturation ≤93% at a rest state; arterial partial pressure of oxygen (PaO2)/oxygen concentration (FiO2) ≤300 mmHg; patients with >50% progression of lesions on lung imaging within 24 to 48 hours should be treated as severe cases. *Critical* cases meet any of the following criteria: occurrence of respiratory failure requiring mechanical ventilation; presence of shock; other organ failure that requires monitoring and treatment in the ICU. In the WHO interim guidance (version 1.2), moderate cases are known as “Pneumonia” while severe cases as “Severe pneumonia”. Critical cases were not available in this cohort.

In this cohort, all the patients were discharged. The criteria for patient discharge was the absence of fever for at least 3 days, substantial improvement in both lungs on chest CT, clinical remission of respiratory symptoms, and two throat-swab samples negative for SARS-CoV-2 RNA obtained at least 24 hours apart^7^. The same cohort of patients was used to investigate non-radiographic risk factors associated with disease severity and length of hospital stay in COVID-19^11^.

### Laboratory procedures

SARS-CoV-2 infection in patients was confirmed using real-time RT-PCR with a standard protocol recommended by China Center for Disease Control and Prevention (CDC). Throat-swab specimens were obtained for examination every other day after admission, but only qualitative data were available. The specimens were considered positive if the *Ct* (cycle threshold) value was ≤37, and negative if the results were undetermined. Specimens with a *Ct* higher than 37 were repeated. The specimen was considered positive if the repeat results were the same as the initial result and between 37 and 40. If the repeat *Ct* was undetectable, the specimen was considered negative.

Routine blood examinations were performed in the same hospital and results were retrieved from electronic medical records.

### High-resolution Computed Tomography (HRCT) scans

All patients underwent chest non-contrast enhanced CT examinations in the supine position and with breath-holding following inspiration. The technical parameters included a 64-section scanner with 1 mm collimation at 5 mm intervals. Images were obtained with both mediastinal (width 350 HU; level 40 HU) and parenchymal (width 1500 HU; level −700 HU) window settings. Frequency of examinations was determined by the treating physician. 11 pneumonia patients were followed up after being discharged,

### Imaging evaluation

For imaging evaluation, 2 experienced chest radiologists reviewed the images independently in a consistent manner, with a final finding reached by consensus when there was a discrepancy. They were blinded to the clinical information or clinical progress of the patients, except for the knowledge that these were cases of COVID-19 patients.

The CT features included ground glass opacity, consolidation, air bronchogram, nodular opacities and pleural effusion. The CT scans were scored on the axial images referring to a method described previously^10^. The extent of involvement of each abnormality was assessed independently for each of 3 zones: upper (above the carina), middle (below the carina and above the inferior pulmonary vein), and lower (below the inferior pulmonary vein). The CT findings were graded on a 3-point scale: normal attenuation (1), ground-glass attenuation (2), and consolidation (3). Each lung zone, with a total of 6 lung zones in each patient, was assigned a following scale according to distribution of the affected lung parenchyma: normal (0), <25% abnormality (1), 25–50% abnormality (2), 50–75% abnormality (3), and >75% abnormality (4). The 4-point scale of the lung parenchyma distribution was then multiplied by the radiologic scale described above. Points from all zones were added for a final total cumulative score (HRCT score), with value ranging from 0 to 72.

### Data collection and sharing

Demographic, clinical, laboratory and radiographic findings were extracted from electronic medical records^10,11^. The data that support the findings of this study are available from the corresponding author upon reasonable request and with permission of Renmin Hospital of Wuhan University, Hubei, China.

### Statistical analysis

Continuous variables were compared with Two Sample *t*-test, Welch Two Sample *t*-test, Mann-Whitney *U* test or Wilcoxon test if appropriate; categorical variables were compared by χ^2^ test or Fisher’s exact test if appropriate. Continuous and categorical variables were expressed as median (interquartile range, IQR) and number (*n*) (%), respectively. Kaplan-Meier plot was used to present viral clearance in COVID-19 patients. The viral clearance day was determined as the median of the last day of viral RNA positive and the first day of viral RNA negative. *P*-values were calculated by log-rank test. Correlations between 2 variables were accessed using Spearman’s Rank-Order correlation. *P*-values less than 0.05 were considered statistically significant. All data analyses and graphs were done in RStudio (V3.6.1) or GraphPad Prism (V8.2.1).

## Results

99 patients (61 pneumonia and 38 severe pneumonia) with key information in their medical records were included in this study.

### Main features in COVID-19 patients with pneumonia or severe pneumonia

The median age of COVID-19 patients with severe pneumonia was 64 years (IQR 57 ~ 69, range 30 to 83), compared with 53 years (IQR 35 ~ 67, range from 24 to 84) in patients with pneumonia (p = 0.0027). The proportion of male patients with severe pneumonia was higher than in the pneumonia patients, although statistical significance was not reached (p = 0.079). Comorbidities were present more frequently in severe pneumonia cases compared to pneumonia (74% *vs*. 34%, p = 0.0003), with hypertension being the most common comorbidity, followed by diabetes and cardiovascular disease. The frequency of COVID-19 patients with hypertension was 50% in cases with severe pneumonia compared with 25% in the pneumonia cases (p = 0.0177) (Table 1).

**Table 1:** Summary of demographic, clinical, and laboratory findings of COVID-19 patients on admission. Highest HRCT score, HRCT peak day and treatments in the clinical course are also included.

	Normal Range	Pneumonia (*n* = 61)	Severe Pneumonia (*n* = 38)	p - value
**Demographics**				
*Age*, *years*		53 (35 ~ 67)	64 (57 ~ 69)	***0.003***
*Sex*				
Male		23 (38%)	22 (58%)	0.079
Female		38 (62%)	16 (42%)	
*Comorbidities*		21 (34%)	28 (74%)	***0.0003***
Hypertension		15 (25%)	19 (50%)	***0.018***
Cardiovascular disease		6 (10%)	4 (11%)	1.0
Diabetes		6 (10%)	9 (24%)	0.19
Cerebrovascular disease		1 (2%)	1 (3%)	1.0
COPD		1 (2%)	1 (3%)	1.0
Asthma		1 (2%)	1 (3%)	1.0
Malignancy		1 (2%)	0 (0%)	1.0
Chronic liver disease		1 (2%)	0 (0%)	1.0
**Signs and Symptoms**				
*Fever*		48 (79%)	34 (89%)	0.267
*Cough*		43 (70%)	33 (87%)	0.103
*Dyspnea*		30 (49%)	29 (76%)	***0.014***
*Anorexia and/or Lethargy*		13 (21%)	19 (50%)	***0.006***
*Fatigue*		15 (25%)	22 (58%)	***0.002***
*Myalgia and/or Arthralgia*		9 (15%)	7 (18%)	0.84
*Diarrhoea*		8 (13%)	4 (11%)	0.76
**Laboratory Findings**				
*White blood cell count* (×10^9^/L)	3.5 ~ 9.5	4.4 (3.35 ~ 5.46)	5.15 (3.80 ~ 6.72)	0.089
*Neutrophil count* (×10^9^/L)	1.8 ~ 6.3	2.73 (1.81~3.78)	3.7900 (2.5575~5.1700)	***0.005***
*Lymphocyte count* (×10^9^/L)	1.1 ~ 3.2	1.23 (0.91 ~ 1.52)	0.775 (0.59 ~ 1.08)	***1.768e-05***
*Neutrophil/Lymphocyte*		2.14 (1.51 ~ 3.43)	4.80 (3.02 ~ 7.60)	***2.237e-06***
*D-dimer* (μg/L)	0 ~ 1	0.35 (0.18 ~ 0.76)	0.67 (0.37 ~ 1.62)	***0.002***
*Lactate dehydrogenase* (U/L)	103 ~ 227	168 (153 ~ 219)	250 (195.75 ~ 360.5)	***3.929e-06***
*C-reactive protein* (mg/dL)	0 ~ 0.6	0.73 (0.33 ~ 2.46)	4.2 (2.47 ~ 7.31)	***1.143e-06***
**Radiographic Findings**				
Highest HRCT score		10 (7 ~ 16)	24.5 (19.0 ~ 31.5)	***3.556e-12***
Peak day		12 (9 ~ 15)	14 (11 ~ 18)	***0.048***

Data are n (%) or median (IQR). p values were calculated by Mann-Whitney U test, χ² test, or Fisher’s exact test, as appropriate. When the data were normally distributed, continuous variables were then described using median and interquartile range (IQR) values. COPD: chronic obstructive pulmonary disease. HRCT: high-resolution computed tomography.

The most common symptoms on admission were fever, cough and dyspnea, followed by fatigue, anorexia and/or lethargy, myalgia and/or arthralgia and diarrhoea. Symptoms such as dyspnea, fatigue, and anorexia and/or lethargy were observed more often in severe pneumonia patients (p < 0.05) (Table 1).

In laboratory findings, lymphocyte counts were significantly decreased whereas neutrophil counts, the neutrophil to lymphocyte ratio (NLR), D-dimer, lactate dehydrogenase and C-reactive protein levels were all increased in severe pneumonia cases (p < 0.05), while there was no significant difference in white blood cell counts (p = 0.089) (Table 1).

**Figure 1:**
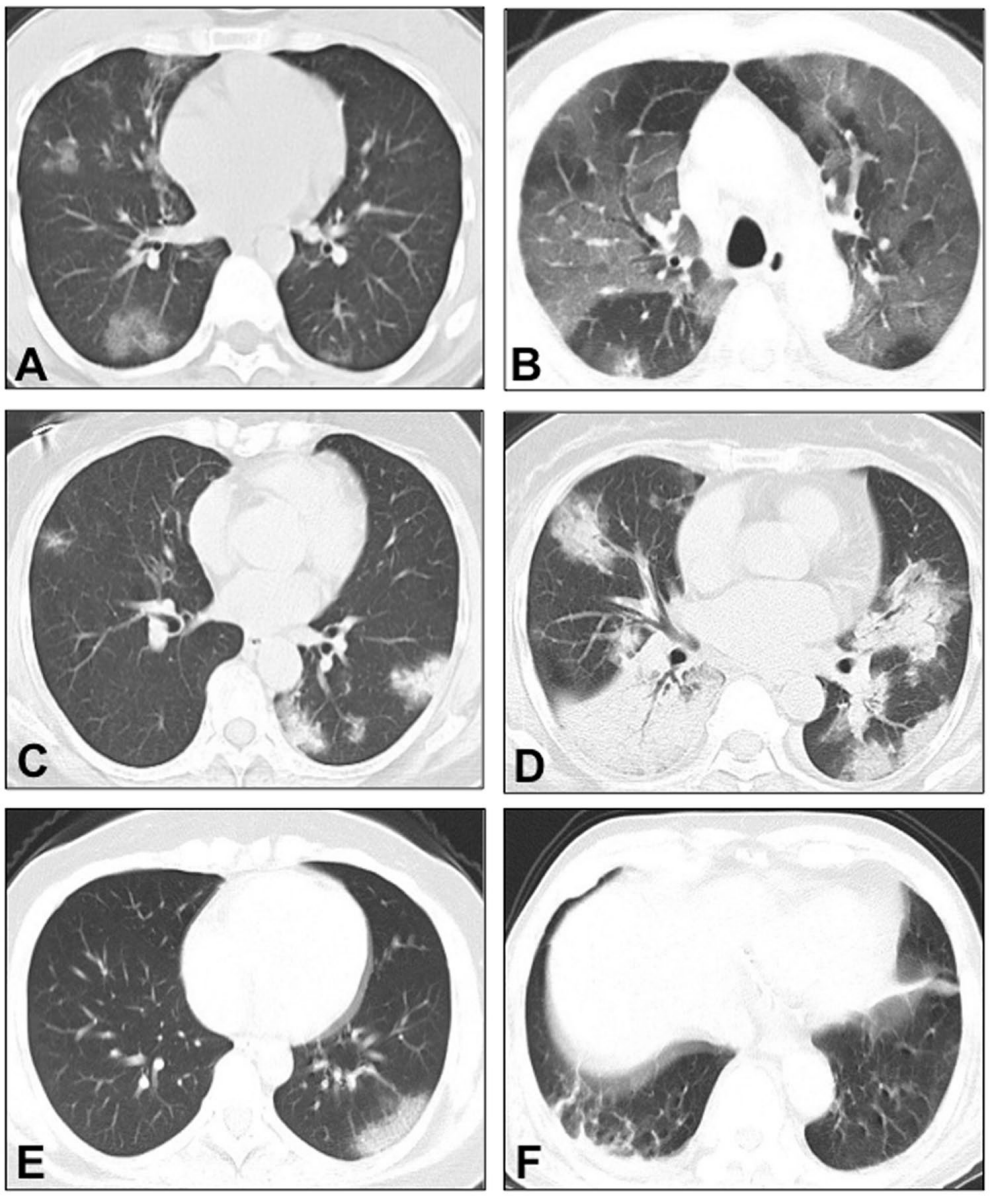
Radiographic features of HRCT scans in patients with confirmed COVID-19. Representative HRCT images showing (**A**) ground glass opacity in a 60-year-old man with pneumonia; (**B**) ground glass opacity and air bronchogram in a 65-year-old man with severe pneumonia; (**C**) consolidation in a 56-year-old woman with pneumonia; (**D**) consolidation and air bronchogram in a 57-year-old woman with severe pneumonia; (**E**) nodular opacities in a 24-year-old woman with pneumonia; (**F**) pleural effusion of the right chest in a 70-year-old man with severe pneumonia.

**Figure 2:**
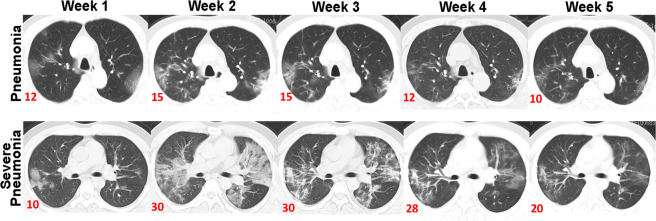
Representative temporal radiographic changes in one pneumonia and one severe case of COVID-19 patient at the indicated week. Numbers in red are HRCT scores.

**Figure 3:**
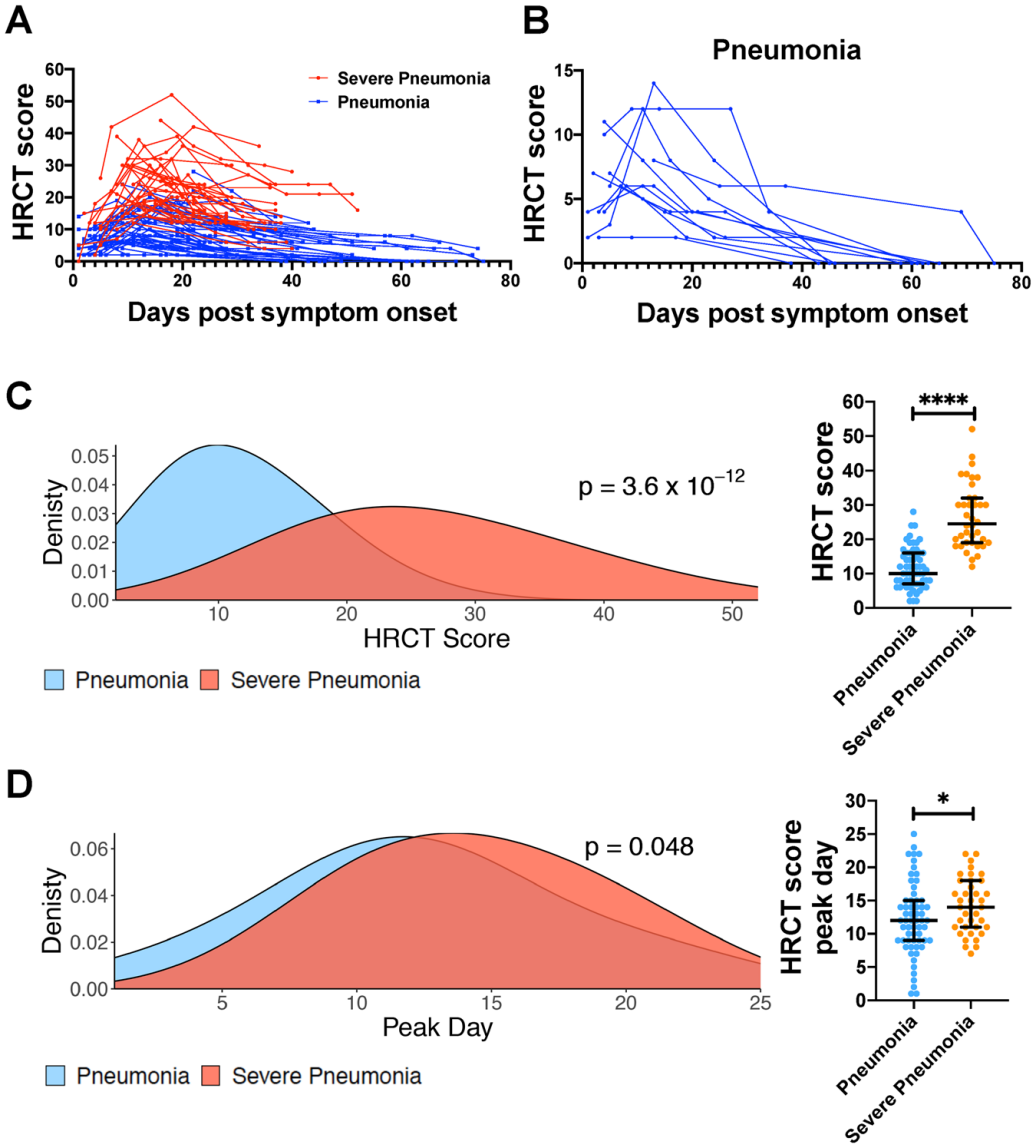
HRCT score and peak day in COVID-19 patients with pneumonia or severe pneumonia. (**A**) Graph showing temporal HRCT score changes in 99 COVID-19 patients with pneumonia (blue) or severe pneumonia (red). (**B**) Graph showing temporal HRCT score changes in 11 pneumonia patients with follow-up CT scans. In (**A**,**B**), each line represents temporal radiographic changes in one COVID-19 patient. Each dot represents a HRCT scan. (**C**) Graphs showing the distributions of HRCT scores (peak) (p = 3.6 × 10^−12^) in COVID-19 patients with pneumonia or severe pneumonia. ****p < 0.0001. (**C**) Graphs showing the distributions of Days to Peak (Peak Day) (p = 0.048) in COVID-19 patients with pneumonia or severe pneumonia. Peak days are the time from admission that it takes for the maximal chest HRCT abnormalities to develop. *p < 0.05.

Radiographic features from HRCT scans included ground glass opacity, consolidation, air bronchogram, nodular opacities and pleural effusion (Fig. 1; Table 2). Temporal radiographic changes in 99 COVID-19 patients were shown in Figs. 2 and 3A. We managed to follow up 11 COVID-19 patients with pneumonia after being discharged, and found their CT scans were all back to normal (Fig. 3B). The HRCT scores (peak) during disease course in COVID-19 patients with severe pneumonia (median: 24.5; IQR range: 19 ~ 31.5) were higher compared to those with pneumonia (median: 10; IQR range: 7 ~ 16) (p = 3.556 × 10^−12^) (Table 1; Fig. 3C), with more frequency of consolidation (84.4% *vs*. 45.9%, p = 0.025) and air bronchogram (50.0% *vs*. 8.2%, p = 7.501 × 10^−6^) (Table 2). The median values of days when the peak HRCT scores were reached in pneumonia or severe pneumonia patients were 12 (IQR range: 9 ~ 15) *vs*. 14 (IQR range: 11 ~ 18), respectively (p = 0.048) (Table 1; Fig. 3D).

**Table 2:** Main HRCT features (peak) in COVID-19 patients with pneumonia or severe pneumonia.

CT Features	Total (*n* = 99)	Pneumonia (*n* = 61)	Severe Pneumonia (*n* = 38)	p - value
*Ground glass opacity*	80 (80.8%)	48 (78.7%)	32 (84.2%)	0.677
*Consolidation*	55 (55.6%)	28 (45.9%)	27 (84.4%)	***0.025***
*Air bronchogram*	24 (24.2%)	5 (8.2%)	19 (50.0%)	***7.501e-06***
*Nodular opacities*	10 (10.1%)	4 (6.6%)	6 (15.8%)	0.176
*Pleural effusion*	11 (11.1%)	5 (8.2%)	6 (15.8%)	0.326

Data are n (%). p values were calculated by χ² test or Fisher’s exact test, as appropriate.

### Relationship between temporal radiographic changes and viral clearance in COVID-19 patients

**Figure 4:**
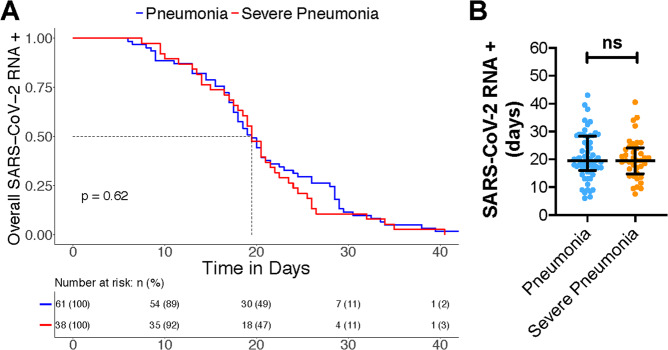
Viral clearance in COVID-19 patients with pneumonia or severe pneumonia. (**A**) Kaplan-Meier plot showing the overall presence of SARS-CoV-2 RNA in pneumonia or severe pneumonia patients. Numbers below are n (%). *P*-value was calculated by log-rank test. (**B**) Graph showing the distributions of SARS-CoV-2 RNA positive days in pneumonia or severe pneumonia patients. ns: not significant. Data are median and IQR.

To check viral clearance in COVID-19 patients, throat-swab specimens were collected to detect SARS-CoV-2 RNA (positive or negative) using real-time RT-PCR routinely. Interestingly, there was no difference in viral clearance in pneumonia *vs*. severe pneumonia patients (Fig. 4A, p = 0.62). The median viral clearance day for pneumonia patients was 21, ranging from 6 to 43; while in severe pneumonia patients, it was 20, ranging from 7.5 to 40.5 (Fig. 4B, p > 0.05). The viral clearance day was determined as the median of the last day of viral RNA positive and the first day of viral RNA negative.

**Figure 5:**
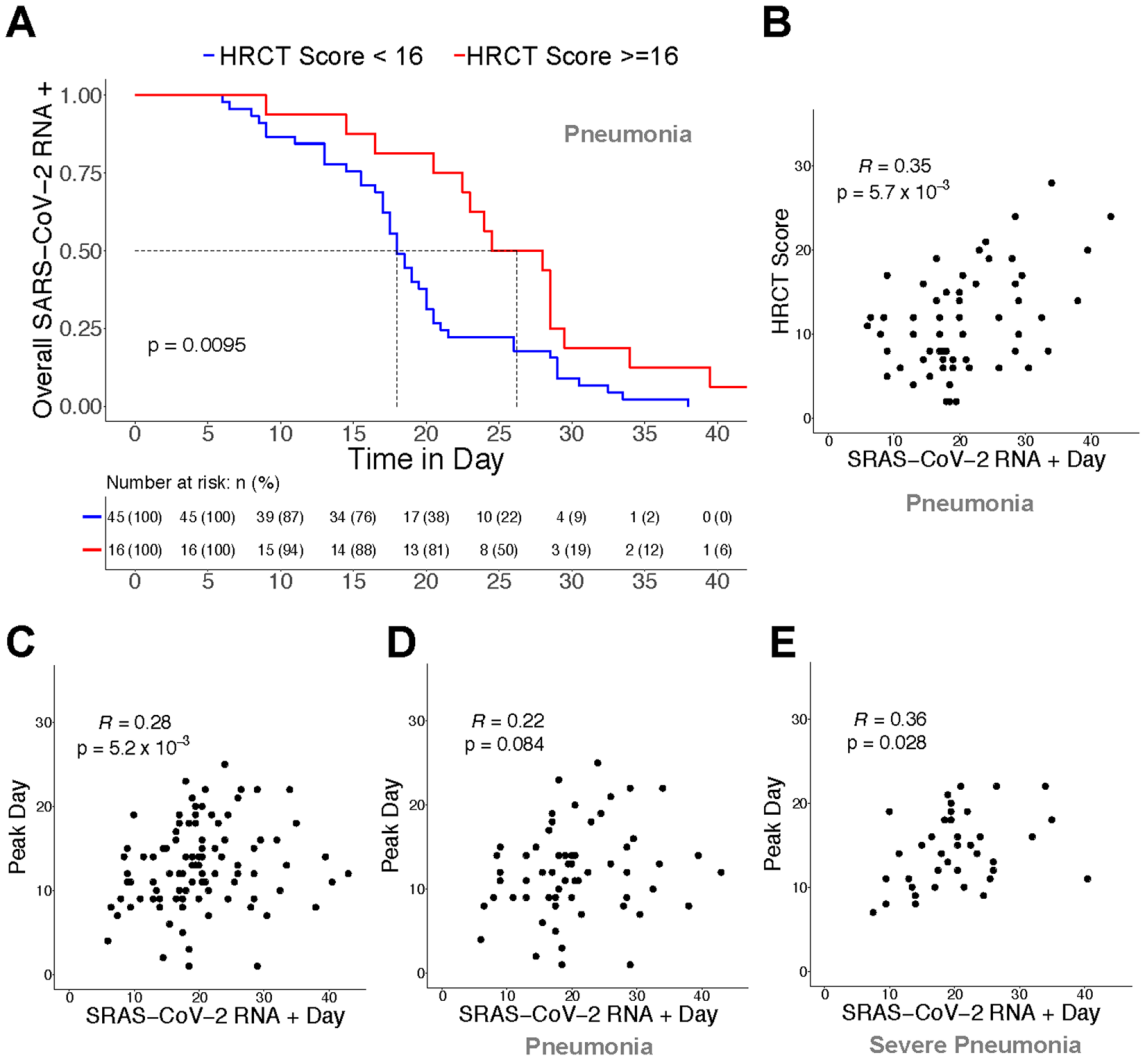
The relationship of HRCT score and peak day to viral clearance. (**A**) Kaplan-Meier plot showing the overall presence of SARS-CoV-2 RNA in pneumonia patients stratified according to the median HRCT score (peak) from all COVID-19 patients in this cohort. Numbers below are n (%). *P*-value was calculated by log-rank test. (**B**) The scatter plot for the correlation between SARS-CoV-2 RNA positive days and HRCT scores (peak) (Spearman’s Rank-Order correlation, *R* = 0.52, p = 5.7 × 10^−3^) in the pneumonia patients. **C–E** The scatter plots for the correlation between SARS-CoV-2 RNA positive days and HRCT score peak day in all (**C**), pneumonia (**D**) or severe pneumonia (**E**) patients, using Spearman’s Rank-Order correlation. Values for *R* and p are included.

We next asked whether there were any demographic, laboratory and radiographic findings associated with viral clearance (summarised in Table 3). We found in COVID-19 patients with pneumonia, HRCT scores (peak) were positively correlated with days to viral clearance using either the log-rank test (Fig. 5A, p = 0.0095) or Spearman’s Rank-Order correlation (Fig. 5B, *R* = 0.35, p = 5.7 × 10^−3^). For the Kaplan-Meier plot of viral clearance, the median HRCT score (16) from all COVID-19 patients in this cohort was used to separate the groups (Fig. 5A). In addition, while the HRCT score peak day was positively correlated with days to viral clearance in all COVID-19 patients (Fig. 5C, *R* = 0.28, p = 5.2 × 10^−3^) and severe pneumonia patients (Fig. 5E, *R* = 0.36, p = 0.028), this failed to reach significance in the pneumonia patients (Fig. 5D, *R* = 0.22, p = 0.084).

**Table 3:** Factors associated with viral clearance in COVID-19 patients.

	p - value		
	All	Pneumonia	Sever Pneumonia
**Demographics**			
*Age*	0.50	0.084	0.28
*Sex*	0.57	0.61	0.98
*Hypertension*	0.26	0.22	0.64
*Cardiovascular disease*	0.76	0.56	0.32
*Diabetes*	0.54	0.15	0.31
**Laboratory Findings**			
*Lymphocyte count*	0.093	0.084	0.41
*White blood cell count*	0.92	0.79	0.42
*Neutrophil count*	0.86	0.89	0.42
*D-dimer*	0.76	0.97	0.66
*Lactate dehydrogenase*	0.54	0.49	0.83
*C-reactive protein*	0.21	0.19	0.98
**Radiographic Findings**			
*Highest HRCT score*	0.079	***5.7e-03***	0.99
*HRCT peak day*	***5.2e-03***	0.084	***0.028***

P values were calculated by log-rank test or Spearman’s Rank-Order correlation, as appropriate.

## Discussion

COVID-19 is “public enemy number one”, says the director general of the WHO. At the time of writing, more than 4.9 million cases have been recorded, with over 320k associated deaths. It is estimated that the case-fatality risk for COVID-19 is in a broad range of 0.25 ~ 3.0%^12^. High mortality rate has been observed in several areas due to over stretched medical resources amongst other things. As a result, understanding the prognostic factors that can predict disease severity will be essential, since this will help frontline clinical staff to stratify patients with increased confidence. In addition, to battle against a highly contagious disease, like COVID-19, elucidating factors that can predict viral clearance is important. In order to provide insight into these questions, we designed a retrospective study, with 99 COVID-19 patients (61 pneumonia and 38 severe pneumonia) with key information from their medical records. Here we report temporal changes in HRCT scores as a valuable predictor for both disease severity and viral clearance.

The features and importance of HRCT scans in the diagnosis of COVID-19 patients have been reported^5,10,13–20^. Similarly, in our cohort, radiographic features included ground glass opacity, consolidation, air bronchogram, nodular opacities and pleural effusion, with more frequency of consolidation and air bronchogram in severe cases, indicating a more severe clinical course for these abnormalities can be pathologically correlated with diffuse alveolar damage^21^. As caused by members within the same virus family, COVID-19 shared common radiographic characteristics with Middle East Respiratory Syndrome (MERS) and severe acute respiratory syndrome (SARS)^22–25^.

Interestingly, with various statistical tools, we were able to show that a higher HRCT score (peak) is strongly correlated with days to viral clearance in COVID-19 patients with pneumonia, while HRCT peak day correlates with days to viral clearance in severe cases. The length of days to viral clearance may be determined by virus load, immune response, treatment and others; while the chest HRCT changes represent the underlying pathophysiology of the disease process. These findings suggest that in COVID-19 patients with pneumonia, damages to the lungs, reflected by HRCT score (peak), are positively correlated to the viral clearance. On the other hand, in severe cases, it is the time taken to develop maximal damage (reflected by HRCT peak day) that positively correlates to the viral clearance. In addition, with follow up CT scans in 11 pneumonia patients after being discharged, we found that pathological changes in the lungs can be completely healed, although more investigation is required in severe cases. Nevertheless, given the values of HRCT scores for both disease severity and viral clearance, a standardised HRCT score system for COVID-19 is thus highly recommended.

There are several limitations in this study. (1) Quantitative viral RNA detection (*Ct* values) was not available. The estimated duration of the presence of viral RNA is limited by the frequency of respiratory specimen collection. (2) Some patients may have received medicine before admission, which may affect chest CT findings. (3) Interpretation of our findings might be limited by the sample size. Despite these limitations, with appropriate statistical tools, we are able to identify the values of monitoring temporal radiographic changes in COVID-19 patients.
